# Supplementary material for: The αC-β4 loop controls the allosteric cooperativity between nucleotide and substrate in the catalytic subunit of protein kinase A
Source: eLife. 2024 Jun 24;12:RP91506. doi: 10.7554/eLife.91506 (PMC11196109; doi:10.7554/eLife.91506)
Supplement: Figure 2—source data 1. [file elife-91506-fig2-data1.docx]

**Figure 2 – source data 1**. ΔG (kcal/mol) and relative population of the ground state and the first 6 excited states in different forms of PKA-C obtained from the RAM simulations.

|  | GS | ES1 | ES2 | ES3 | ES4 | ES5 | ES6 |
| --- | --- | --- | --- | --- | --- | --- | --- |
| Apo | 0  (58.0%) | 0.38 (30.8%) | 1.22  (7.6%) | 2.10  (1.8.%) | 2.28  (1.3%) | 2.85  (0.5%) | 8.16*  (<1e-4) |
| Binary | 0  (99.4%) | 3.11  (0.6%) | 5.80  (<1e-4) | 6.66  (<1e-4) | 7.25  (<1e-4) | 8.27  (<1e-4) | 8.47  (<1e-4) |
| Ternary | 0  (100.0%) | 4.85  (<1e-4) | 5.68  (<1e-4) | 6.92  (<1e-4) | 7.04  (<1e-4) | 7.58  (<1e-4) | 7.87  (<1e-4) |

* Numbers in red refer to populations of excited states below 0.5%.
