# Supplementary material for: The αC-β4 loop controls the allosteric cooperativity between nucleotide and substrate in the catalytic subunit of protein kinase A
Source: eLife. 2024 Jun 24;12:RP91506. doi: 10.7554/eLife.91506 (PMC11196109; doi:10.7554/eLife.91506)
Supplement: Supplementary file 1. — The KM and Vmax values were obtained from a nonlinear least squares analysis of the concentration-dependent initial phosphorylation rates. Errors in the kcat/KM ratios were propagated from the individual errors in KM and kcat. [file elife-91506-supp1.docx]

**Supplementary file 1. Kinetic parameters of Kemptide phosphorylation by PKA-C^WT^ and PKA-C^F100A^ obtained from coupled assays.** The *K_M_* and *V_max_* values were obtained from a nonlinear least squares analysis of the concentration-dependent initial phosphorylation rates. Errors in the *k_cat_*/*K_M_* ratios were propagated from the individual errors in *K_M_* and *k_cat_*.

|  | **PKA-C^WT^** | **PKA-C^F100A^** |
| --- | --- | --- |
| *V_max_* | 0.322 ± 0.005 | 0.379 ± 0.009 |
| *K_M_* | 30 ± 1 | 42 ±3 |
| *k_cat_* | 15 ± 1 | 17 ± 1 |
| *k_cat_*/*K_M_* | 0.50 ± 0.04 | 0.41 ± 0.08 |
