# Supplementary material for: The αC-β4 loop controls the allosteric cooperativity between nucleotide and substrate in the catalytic subunit of protein kinase A
Source: eLife. 2024 Jun 24;12:RP91506. doi: 10.7554/eLife.91506 (PMC11196109; doi:10.7554/eLife.91506)
Supplement: Supplementary file 2. — All errors were calculated from triplicate measurements. Values for PKA-CWT are taken from Walker et al., 2019 [file elife-91506-supp2.docx]

**Supplementary file 2. Changes in enthalpy, entropy, free energy, and dissociation constants for nucleotide binding to PKA-C^WT^ and PKA-C^F100A^.** All errors were calculated from triplicate measurements. Values for PKA-C^WT^ are taken from Walker *et al.* ^22^

|  | *K*_d_ (μM) | ΔG (kcal/mol) | ΔH (kcal/mol) | -TΔS (kcal/mol) | σ |
| --- | --- | --- | --- | --- | --- |
| PKA-C^WT^ | 83 ± 8 | -5.61 ± 0.06 | -3.6 ± 0.1 | -2.0 ± 0.1 | N/A |
| PKA-C^F100A^ | 73 ± 2 | -5.7 ± 0.2 | -21 ± 5 | 7 ± 3 | N/A |
