# Supplementary material for: The αC-β4 loop controls the allosteric cooperativity between nucleotide and substrate in the catalytic subunit of protein kinase A
Source: eLife. 2024 Jun 24;12:RP91506. doi: 10.7554/eLife.91506 (PMC11196109; doi:10.7554/eLife.91506)
Supplement: Supplementary file 3. — All errors were derived from triplicate measurements. The error for the cooperativity coefficient (σ) was propagated from the errors in Kd. Values for PKA-CWT were originally published in Walker et al., 2019. [file elife-91506-supp3.docx]

**Supplementary file 3. Changes in enthalpy, entropy, free energy, and dissociation constants for PKI_5-24_ binding to apo and ATPγN - saturated PKA-C^WT^ and PKA-C^F100A^.** All errors were derived from triplicate measurements. The error for the cooperativity coefficient (σ) was propagated from the errors in *K*_d_. Values for PKA-C^WT^ were originally published in Walker *et al.* ^22^.

| PKI_5-24_ to apo kinase | | | | | |
| --- | --- | --- | --- | --- | --- |
|  | *K*_d_ (μM) | ΔG (kcal/mol) | ΔH (kcal/mol) | -TΔS (kcal/mol) | σ |
| PKA-C^WT^ | 17 ± 2 | -6.57 ± 0.08 | -10.8 ± 0.5 | 4.2 ± 0.5 | N/A |
| PKA-C^F100A^ | 5 ± 1 | -7.7 ± 0.1 | -17 ± 5 | 7 ± 3 | N/A |

| PKI_5-24_ to ATPγN-saturated kinase | | | | | |
| --- | --- | --- | --- | --- | --- |
|  | *K*_d_ (μM) | ΔG (kcal/mol) | ΔH (kcal/mol) | -TΔS (kcal/mol) | σ |
| PKA-C^WT^ | 0.16 ± 0.02 | -9.33 ± 0.07 | -13.9 ± 0.5 | 4.6 ± 0.4 | 106 ± 18 |
| PKA-C^F100A^ | 2 ± 1 | -7.9 ± 0.3 | -17 ± 1 | 9 ± 1 | 3 ± 1 |
